# Repair of Infected Bone Defects with Hydrogel Materials

**DOI:** 10.3390/polym16020281

**Published:** 2024-01-19

**Authors:** Zhenmin Cao, Zuodong Qin, Gregory J. Duns, Zhao Huang, Yao Chen, Sheng Wang, Ruqi Deng, Libo Nie, Xiaofang Luo

**Affiliations:** 1 Hunan Key Laboratory of Biomedical Nanomaterials and Devices, Hunan University of Technology, Zhuzhou 412007, China; czm320721@163.com (Z.C.); qinzd@huse.edu.com (Z.Q.); huangzhao2519@126.com (Z.H.); chenyao717@hnu.edu.cn (Y.C.); 18305856170@163.com (S.W.); dengruqi377@163.com (R.D.); 2 Hunan Engineering Technology Research Center for Comprehensive Development and Utilization of Biomass Resources, College of Chemistry and Bioengineering, Hunan University of Science and Engineering, Yongzhou 425199, China; gjduns@gmail.com

**Keywords:** hydrogels, infected bone, antimicrobial, bone regeneration

## Abstract

Infected bone defects represent a common clinical condition involving bone tissue, often necessitating surgical intervention and antibiotic therapy. However, conventional treatment methods face obstacles such as antibiotic resistance and susceptibility to postoperative infections. Hydrogels show great potential for application in the field of tissue engineering due to their advantageous biocompatibility, unique mechanical properties, exceptional processability, and degradability. Recent interest has surged in employing hydrogels as a novel therapeutic intervention for infected bone repair. This article aims to comprehensively review the existing literature on the anti-microbial and osteogenic approaches utilized by hydrogels in repairing infected bones, encompassing their fabrication techniques, biocompatibility, antimicrobial efficacy, and biological activities. Additionally, the potential opportunities and obstacles in their practical implementation will be explored. Lastly, the limitations presently encountered and the prospective avenues for further investigation in the realm of hydrogel materials for the management of infected bone defects will be deliberated. This review provides a theoretical foundation and advanced design strategies for the application of hydrogel materials in the treatment of infected bone defects.

## 1. Introduction

Bone tissue infections are lesions of bone tissue caused by microbial infections. Common sources include surgical wound infections, trauma, and osteoclast infections, which are common orthopedic complications [1,2]. Despite notable advancements in medical technology, the clinical management of antimicrobial treatment and osteogenic regeneration for infected bone defects continues to pose challenges [3]. The conventional approach to addressing infected bone defects typically involves bone grafting and antibiotic therapy [4]. The utilization of bone grafting with autologous material in the medical field necessitates supplementary surgical procedures due to limited resources. However, this approach is associated with potential complications such as immune rejection, donor site morbidity, and stress fractures [5,6]. Furthermore, the long-term administration of systemic antibiotics is susceptible to heightened antibiotic resistance and adverse health effects. In recent years, tissue engineering using biological substitutes to replace or repair damaged tissue has emerged as a noteworthy treatment option due to its promising application potential [7,8,9].

A successful treatment for infected bone defects should resolve bacterial infection control and bone defect repair simultaneously. Achieving this requires the utilization of appropriate biomaterials capable of controlled drug release and restorative therapy. In a series of biological materials, hydrogels stand out not only for treating lesions but also for facilitating the repair of structural defects [10]. Hydrogels can be finely adjusted through chemical modifications to attain desirable degradability and mechanical stability. Hydrogels can replicate dynamic interactions in natural environments through the implementation of sustainable dynamic chemistry. Above all, hydrogels can be conjugated with cells, growth factors, or pharmaceutical agents to establish an environment conducive to cell infiltration, adhesion, growth, proliferation, migration, and differentiation [11,12,13,14]. As a result, hydrogels are widely used in various fields such as medicine and bioengineering [15].

There are typically two categories of hydrogels based on the origin of the gel material, namely, synthetic polymer hydrogels and natural polymer hydrogels [16]. Synthetic polymer hydrogels exhibit favorable water-absorbing capabilities and effective stability. However, synthetic polymer hydrogels are complicated and costly to synthesize and may cause allergic reactions. It is difficult to adjust the modulus of elasticity to suit specific microtissue environments, limiting its application [17]. Traditional hydrogel forming techniques with fixed shapes are difficult to adapt to irregular injury repair, such as bone repair hydrogels that rely on mold formation. The application is limited by its inefficiency in treating large complex bone defects and its inability to adequately fill irregular defects [18]. Injectable hydrogels are more suitable for the repair of irregular injuries [19]. Conversely, natural materials offer numerous benefits such as inherent biocompatibility and degradability, along with a diverse array of macromolecular designs that facilitate versatile structural applications [20,21]. Hydrogels facilitate the efficient execution of their intended functions by creating a suitable space and microenvironment [22]. The hydrogel also encapsulates drugs and other therapeutic ingredients within its structure for in situ delivery to the infected area for long-term effective treatment [23,24]. The formation of stimuli-responsive hydrogels by manipulation of polymer molecular chains enables intelligent drug release [25,26,27]. With the degradation of the hydrogel scaffold, cells can proliferate, differentiate, and secrete extracellular matrix material, ultimately facilitating functional bone reconstruction [28].

This paper presents a comprehensive examination of the utilization of hydrogels for the purpose of repairing infected bone defects. The discussion encompasses an analysis of hydrogel composition and structure, as well as an exploration of the diverse functional properties that hydrogels can attain. Particular emphasis is placed on the development of hydrogels specifically tailored for the repair of infected bone defects, encompassing the selection of suitable materials (both natural and synthetic) and the implementation of infection control measures such as antibiotics, nanoparticles, and antimicrobial peptides (AMPs). The focus is a summary on loading different antimicrobial agents in hydrogels for bone repair along with antimicrobial properties. As well as hydrogels to control the release of bone repair drugs. It has a certain antibacterial effect while enhancing bone repair. A comprehensive overview of the application of multiple drug-loading systems of hydrogels in infected bone repair can provide a theoretical basis for the development of hydrogel repair of infected bone defects. Additionally, the challenges associated with the application process of hydrogels are thoroughly examined. Furthermore, the prospects for incorporating hydrogel materials in bone tissue engineering are envisioned. Ultimately, this review aims to contribute to the advancement of hydrogel-based therapies for the treatment of bone infections in the foreseeable future.

## 2. Hydrogel Materials

### 2.1. Definition

The majority of hydrogels consist of one or more polymers interconnected within an insoluble hydrophilic copolymer network, forming a three-dimensional framework capable of absorbing and retaining water for extended periods [29]. Hydrogels exhibit exceptional water absorption properties, enabling them to absorb multiple times their own weight in water. Upon water absorption, a gel is formed, maintaining structural integrity and resisting water loss. The mechanical strength of hydrogels can be modulated by several factors. The manipulation of the physical cross-linking structure, and the control of the cross-linking density ensure a stable three-dimensional framework. The presence of hydrophilic groups (e.g., amino, carboxyl, and hydroxyl) helps to enhance the unique water retention capacity of hydrogels [30,31]. By considering these factors, hydrogels can be tailored to exhibit desired structural characteristics and water retention properties, while also allowing for control over their internal transport and diffusion attributes. Consequently, hydrogels are commonly employed in the medical domain for applications such as wound dressings, tissue engineering, and drug delivery [32]. By virtue of their ability to absorb blood and secretions from injured regions, hydrogels facilitate wound cleanliness and contribute to wound healing. Additionally, their biocompatibility, biodegradability, and high porosity make them efficacious in promoting new bone generation, while the encapsulation of drugs within the gel matrix enables sustained release, thereby yielding a prolonged therapeutic effect [33,34,35].

### 2.2. Classification

**Figure 1:**
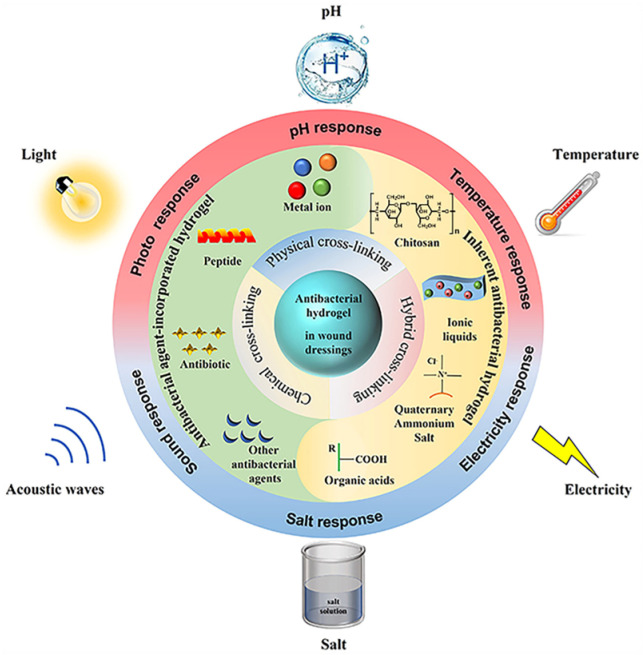
Crosslinking processes for preparation and various applications of hydrogels. Reproduced from ref. [37]. Copyright 2023, with permission from Elsevier.

Hydrogels are primarily classified based on the origin of the polymer, which can be distinguished as either natural or synthetic. The formation of a hydrogel polymer involves both chemical and physical cross-linking processes [36,37] (Figure 1). Chemical cross-linking entails the covalent bonding of precursor functional groups to generate larger copolymer molecules. This covalent linkage between polymer molecules imparts enhanced mechanical strength to hydrogels [38]. Physically crosslinked hydrogels are easy to synthesize because they do not require crosslinking agents but are formed using ionic, electrostatic, and hydrophobic interactions [39]. Natural polymers demonstrate exceptional biocompatibility, biodegradability, and a diverse array of macromolecular configurations that facilitate cell adhesion [40]. Conversely, synthetic hydrogels offer the ability to modify various fundamental structural components, resulting in products with distinct porosity, degradation rates, and mechanical characteristics. Additionally, these hydrogels possess low immunogenicity, enabling the design of preparation strategies tailored to specific requirements [41]. Most conventional hydrogels can only achieve stimulus responsiveness by altering their swelling state and unrestricted diffusion of components. Specific hydrogels, on the other hand, can achieve this property by changing their material and structural characteristics. Consequently, these hydrogels may acquire multiple functionalities, thereby broadening their potential application in infected bone therapy [42]. 

#### 2.2.1. Natural Hydrogels

Natural hydrogels are hydrogels whose raw materials are based on natural biological materials, including proteins and polysaccharides [22]. Natural polymers consist of natural constituents found in living organisms and exhibit both biological and chemical resemblances to natural tissues. These hydrogels possess favorable biocompatibility, inducing minimal immune response and cytotoxicity. Due to their striking resemblance to the extracellular matrix, natural hydrogels serve as an exceptional biomimetic matrix material for bone tissue engineering. This characteristic facilitates cell adhesion, proliferation, and the regeneration of new tissues [43,44]. However, the mechanical properties of natural hydrogels prepared by traditional methods are generally poor. The mechanical properties of natural hydrogels needs to be improved to enhance osteoinductivity [45]. Hence, modification to natural hydrogels or the introduction of other materials is necessary to form scaffold materials that are more appropriate for bone tissue engineering.

Chondroitin sulfate, a crucial constituent of the extracellular matrix, exerts regulatory effects on bone remodeling within the intraosseous microenvironment by mediating the differentiation of osteoclasts and osteoblasts. However, it has the limitation of high solubility under physiological conditions [46]. Guan-synthesized three-dimensional hydrogels encapsulating bone marrow mesenchymal stem cells (BMSCs) use oxidized chondroitin sulfate via dynamic Schiff base bonding. The transformation of BMSCs into chondrocytes and the repair of damaged growth plates were facilitated [47]. Hao et al. demonstrated that chondroitin sulfate is involved in biomineralization in its free state which promotes bone tissue repair [48]. Hyaluronic acid (HA) is a naturally occurring hydrophilic, non-immunogenic glycosaminoglycan that supports bone growth when combined with osteoconductive molecules. It has advantages in tissue healing and angiogenesis, but usually requires modification to form stable hydrogels [49]. Choi et al. added inorganic minerals to HA hydrogels. The structural and mechanical properties of the hydrogel were improved to stimulate the formation of new bone at the cranial defects [50]. Chitosan (CS) hydrogel has antimicrobial properties, easy sterilization, biocompatibility, and controlled degradation. However, it has poor mechanical properties that can be overcome by cross-linking with other materials [51]. Sodium alginate (SA) hydrogels are biocompatible but have poor cell adhesion and poor mechanical properties [52]. Porosity and degradation time will affect the controlled release of drugs from the hydrogel as well as bone replacement as the repair proceeds. The optimization of hydrogel mechanical properties is crucial for their suitability in supporting the mechanical demands of daily exercise for bone regeneration. Therefore, hydrogels must exhibit stiffness, toughness, and swelling stability under physiological conditions. In dual-network hydrogels, there is usually a rigid network that has a slight cross-linking, and a layer network that has a strong cross-linking, resulting in good mechanical properties [38]. For example, Guo et al. conducted a study in which alkaline phosphatase (ALP) was mixed with a solution of SA-polyacrylamide (SA-PAM) precursor using *N*,*N*′-methylene-bisacrylamide and ammonium persulfate to create the network structure of AM. Calcium, strontium, and zinc ions were then introduced to form the network structure of SA. The resulting hydrogel was subsequently immersed in calcium glycerophosphate for mineralization. This hydrogel exhibited improved compressive and tensile mechanical properties, swelling stability, and cellular response, which can be attributed to the combined effects of ionic cross-linking and enzymatic mineralization [53].

#### 2.2.2. Synthetic Hydrogels

Synthetic materials such as polyvinyl alcohol (PVA), polycaprolactone (PCL) and polyethylene glycol (PEG) can be used for the synthesis of bone repair hydrogels [54]. Although synthetic materials may not exhibit the same level of biocompatibility and biological properties as natural materials, their inherent structural units allow for the customization of porosity, degradation time, and mechanical properties to cater to specific application requirements [55]. PVA hydrogels are biocompatible and have good water solubility and biodegradability but pure PVA hydrogels have poor mechanical properties [56]. PCL hydrogels have good biodegradability, biocompatibility and non-toxicity, but lack mechanical strength [57]. PCL hydrogel is elastic, thermally stable, flexible and biocompatible. However, its slow degradation rate makes it suitable for application as a long-term implant material. For example, PCL compounded with collagen has improved mechanical properties. Moreover, composite scaffolds can induce osteogenic differentiation of adipose mesenchymal stem cells, which has the potential for bone regeneration applications [58]. PEG hydrogels are biocompatible, resistant to cellular protein adhesion and have low toxicity but have moisture sensitivity. Roumani et al. conducted a study in which they developed hydrogels based on L-lysine dendrimer graft (DGL) and PEG with adjustable polymerization of reverse porosity. This modified hydrogel demonstrated the ability to enhance the interaction between endothelial cells and hMSCs, thereby increasing osteogenic potential [59]. Synthetic polymer hydrogels show great promise as carriers for delivering active proteins, growth factors, and drugs to bone tissue. In a similar vein, Xue et al. employed CuS nanoparticles to cross-link PEG hydrogels, resulting in hydrogel scaffolds with exceptional photothermal and mechanical properties. CuS-PEG-PCL scaffolds could release dexamethasone sodium phosphate (Dexp) when irradiated with near-infrared light at 1064 nm. This approach effectively induced the differentiation of bone mesenchymal stem cells into osteogenic cells [60]. However, the challenges of synthesizing polymer hydrogels as drug carriers still need to be faced. There is a need to optimize the drug delivery system to control its slow release to the target, helping the drug to regulate its biological activity safely over a longer period to generate biologically active tissues. The advantages and limitations of hydrogel materials can be seen in Table 1.

### 2.3. Preparation and Application of Hydrogels

Hydrogels are usually prepared using hydrophilic monomers to form a cross-linked network capable of absorbing water [29]. Most natural hydrogels are formed through a physical cross-linking process. Hydrogels are formed using changes in intermolecular interactions such as hydrogen bonding gelation, hydrophobic interactions, and ionic cross-linking. SA hydrogels are representative of ionic cross-linked hydrogels. Under vigorous stirring, the appropriate amount of metal ions can be uniformly coordinated with the gluconic acid portion of the SA molecule through a homogeneous unsaturated cross-linking strategy to form SA hydrogels. This approach avoids the localized excessive cross-linking caused by traditional drop and soak methods [62]. In addition, a dual network structure was formed by repeated freeze–thawing of PVA and cross-linking of SA ions, followed by enzyme mineralization. The enzyme mineralization and dual network structure preparation improved the hydrogel’s mechanical properties. The combination of the biological properties, strength and toughness, shows great potential for bone tissue engineering applications [63]. Carboxymethyl CS is affixed with α-cyclodextrin (α-CD), where the conjugation of α-CD improves water-soluble and hydrophobic cavities, thus acting as a cross-linking point. An injectable CS-based hydrogel for bone tissue engineering was formed [64]. Synthetic hydrogels such as PCL polymers have been recognized and approved by the FDA for numerous biomedical applications [65]. Rapid photocrosslinking gelation of methacrylate CS under 405 nm light irradiation. It can also be combined with dynamic imine bonds from Schiff base reactions between aldehydes and polyethylene glycols and can be rapidly formed in situ for intervertebral disc repair injectable CS/PEG hydrogels [66]. Preparation of injectable thermosensitive hydrogels based on polyacrylamide polyacrylic acid copolymers can be formed by cross-linking two precursor solutions of significantly different viscosities. The monomer, crosslinker and initiator are dissolved in water to form a lower viscosity solution A. Superimposed on a higher viscosity precursor solution B consisting of the same/different monomer and additional macromolecules. The high viscosity contrast between the two solutions will limit the diffusion of monomers to maintain the bilayer structure, while some limited substances (monomers, cross-linkers) still exchange at the interface to form an interpenetrating network. The prepared hydrogels are able to be applied to soft actuators, wound healing patches and wearable electronic devices [67].

## 3. Antibacterial Hydrogels for Bone Infection Repair

### 3.1. Release-Based Antimicrobial Hydrogel

The treatment and regeneration of infected bone defects pose weighty challenges in clinical practice due to the complexities associated with antimicrobial therapy [68,69]. Bacterial invasion of bone tissue triggers the secretion of acidic metabolites and elicits a robust immune response, thereby diminishing osteoblast activity and impeding the repair process of bone defects [70]. Concurrently, bacteria produce pathogenic and cytotoxic factors through toxin secretion, resulting in detrimental effects on the bone matrix, necrosis of nerves and blood vessels, and substantial hindrance to the regeneration of bone [71]. As a result, there is a growing interest in antimicrobial hydrogels within the field of bone tissue engineering. These hydrogels, utilized for bone repair, exhibit the ability to accurately and efficiently eliminate bacteria through the incorporation of diverse antimicrobial agents such as antibiotics, metal nanoparticles, and AMPs [72,73,74]. Furthermore, the gradual release of these antimicrobial agents from the hydrogels contributes to their notable bacteriostatic effect, rendering them effective in infection control and the prevention of postoperative infections.

#### 3.1.1. Antibiotics

Medical implants and devices employed in orthopedic prosthetic surgery exhibit a high susceptibility to infections which poses challenges in terms of control. The conventional approach to treatment involves systematic administration of antibiotics, yet this has proven to be largely ineffective and may contribute to the emergence of bacterial resistance and associated health risks [75,76]. Commonly utilized antibiotic-loaded materials, such as bone cement, serve as a means of locally releasing antibiotics to combat infections. However, these materials are accompanied by drawbacks such as abrupt antibiotic release and the need for subsequent surgical removal [77]. Research advancements have led to the development of multifunctional hydrogels capable of loading antibiotics, thereby serving as a preventive measure against surgical infections and aiding in infection control. These hydrogels possess the ability to release antibiotics directly at the site of infection, thereby effectively preventing and controlling infections. For example, rifampicin, a widely employed RNA polymerase blocker, is commonly utilized for the treatment of bone infections. Yuan et al. used guar gum/CS/polycaprolactone to formulate the interconnected micellar core of the hydrogel into a hydrophilic inner core and a hydrophobic outer core, which overcame the problem of poor water solubility of rifampicin while also possessing excellent antimicrobial activity [78]. Gao et al. conducted a study wherein vancomycin (Van)-based CS hydrogels were utilized on polylactic acid (PLA)/nanohydroxyapatite (nHAP) scaffolds with staggered and vertical orthogonal structures. This study successfully developed a system for localized antibiotic release. The composite scaffolds exhibited sustained release for a duration of over 8 weeks in vitro, and the materials demonstrated biocompatibility by not negatively impacting the proliferation or differentiation of mouse embryonic osteoblasts [79].

**Figure 2:**
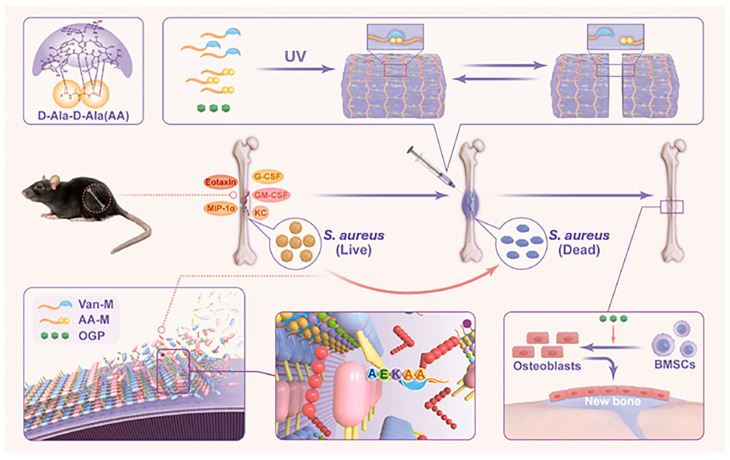
Synthesis and application of Van-AA-OGP hydrogels and mechanism of antimicrobial and osteogenic action. The recognition of Van and specific terminal AA dipeptide of the cell wall peptidoglycan precursor in nature. The synthesis and self-healing characteristics of hematoma-like Van-AA-OGP hydrogel. Van-M (marked blue) and AA-M (marked yellow) paired in the hydrogel demonstrate the reversible Van–AA interaction. Van-AA-OGP hydrogel eliminates *S. aureus* and promots bone repair in murine fracture infection. Reproduced from ref. [83]. Copyright 2023, with permission from Elsevier.

Methicillin-resistant Staphylococci poses weighty challenges in the context of bone infections and their treatment, particularly in cases of chronic bone infections, often resulting in impaired bone healing and inflammatory responses. In a study conducted by Yu et al., a combination therapy involving Van-rifampicin-trametinib antibiotics was employed to address impaired bone fracture healing in the presence of MRSA infections. This integrated approach, which incorporated adjunctive cell-penetrating antibiotic therapy, successfully mitigated excessive inflammation and promoted the healing process in infected fractures [80]. In addition, Motasadizadeh et al. developed filipin/SA hydrogels containing a dual drug load of ticlopidine and fenarimide with sustained and pH-sensitive release behavior and a higher release rate at alkaline pH. Infected bone treated with hydrogel scaffolds effectively inhibited methicillin-resistant *Staphylococcus aureus* infection and had higher bone regeneration. It may be possible to treat chronic bone infections effectively with this dual-drug delivery system [81]. Moreover, Yao et al. developed a pH-sensitive gel containing gelatin methacryloyl (GelMA) and oxidized sodium alginate (OSA) to deliver gentamicin sulfate (GS) and benzylamine, which enabled the hydrogel to have both antimicrobial and osteoinductive properties [82]. Wang et al. proposed a dynamic hydrogel network to reversibly recognize Van and its target dipeptide D-Ala-D-Ala (AA), along with the introduction of endogenous osteogenic peptide (OGP), which can rapidly clear bacteria and promote bone repair [83] (Figure 2).

#### 3.1.2. Metal Nanoparticles

**Figure 3:**
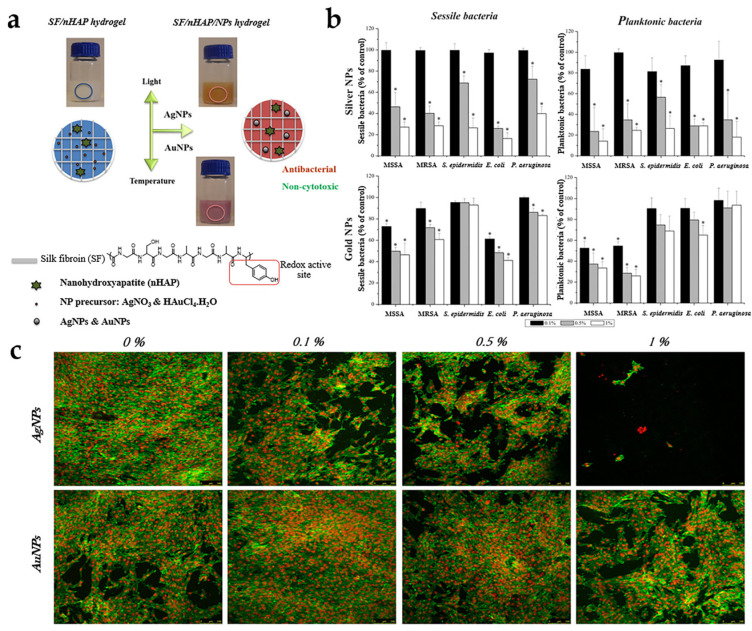
(**a**) SF/nHAP hydrogel preparation scheme; (**b**) sessile and planktonic growth of MSSA, MRSA, *S. epidermidis*, *E. coli* and *P. aeruginosa* on SF/nHAP hydrogels containing different AgNPs and AuNPs concentrations, as percentages of the control materials without nanoparticles, after 24 h of incubation. * *p* < 0.01, significant reduction compared to hydrogels without NPs; (**c**) CLSM images of osteoblastic cells at day 7 on SF/nHAP hydrogels with different concentrations of AgNPs and AuNPs. MG63 cells were stained for F-actin cytoskeleton with alexafluor phalloidin (green) and nuclei with propidium iodide (red). Reproduced from ref. [90]. Copyright 2017, with permission from Elsevier.

Metal nanoparticles such as silver, gold, and zinc oxide, exhibit distinctive optical and electronic characteristics alongside antibacterial properties [84,85,86]. The bactericidal mechanism of nanosilver is mainly to destroy the outer membrane of bacteria and generate reactive oxygen species (ROS). It has the advantage of comprehensive sterilization and can be widely used in major devices in the antibacterial field [87]. Qiao et al. conducted a study in which silver nanoparticles were implanted into a 3D-printed porous titanium scaffold system. Prior to implantation, the nanoparticles were incorporated into a supramolecular hydrogel that possessed effective antimicrobial properties. This approach not only facilitated bone repair and osseointegration in infections but also demonstrated promising potential for future applications in the field of medicine [88]. Similarly, Ou et al. explored the incorporation of nanosilver into eclogite nanotube/gelatin methacrylate hybridized hydrogels. This integration allowed for the utilization of halloysite nanotubes (HNTs) and their exceptional drug delivery and bone regeneration capabilities. The resulting hybrid hydrogels exhibited long-term broad-spectrum antimicrobial activity, attributed to the strong electrostatic adsorption of nanosilver and also to the effective anti-inflammatory effects [89]. In addition, the utilization of multiple metal nanoparticles in conjunction with hydrogels can be employed to augment the antimicrobial characteristics of hydrogel materials. Ribeiro et al. introduced a remarkably eco-friendly, expeditious, and straightforward method for producing antimicrobial silk fibroin (SF)/nHAP hydrogels, wherein SF serves as both a matrix and a reducing agent. This method leads to the synthesis of silver and gold nanoparticles (AuNPs) that are evenly dispersed. Tyrosine, owing to its electro-donating attributes, emerges as one of the most accessible potent electron donors. When combined with its broad-spectrum antimicrobial activity, the developed materials exhibit biological properties that render them suitable for mending bone defects [90] (Figure 3).

In addition to silver nanoparticles, zinc oxide nanoparticles (ZnO NPs) have emerged as a promising class of inorganic nanomaterials with antimicrobial properties in the field of biomedicine. El-Naggar et al. developed a nanogel composed of carboxymethyl cellulose and PEG incorporating ZnO NPs. Carboxymethyl cellulose was employed as a stabilizer to facilitate the preparation of solid ZnO NPs. The inclusion of a high concentration of ZnO NPs not only reduced the surface area and swelling activity, but also enhanced the hydrothermal stability of the hydrogel, resulting in notable antimicrobial activity [91]. In a study conducted by Christy et al., it was observed that the incorporation of ZnO NPs into CS/PVA hydrogels resulted in enhanced tensile strength, apparent density, and improved inhibition of Gram-positive bacteria [92]. These findings suggest that the integration of metal nanoparticles into hydrogels not only optimizes their structural properties but also enhances their antimicrobial efficacy, thereby offering weighty potential for regenerative repair of infected bone defects.

#### 3.1.3. Antimicrobial Peptides

**Figure 4:**
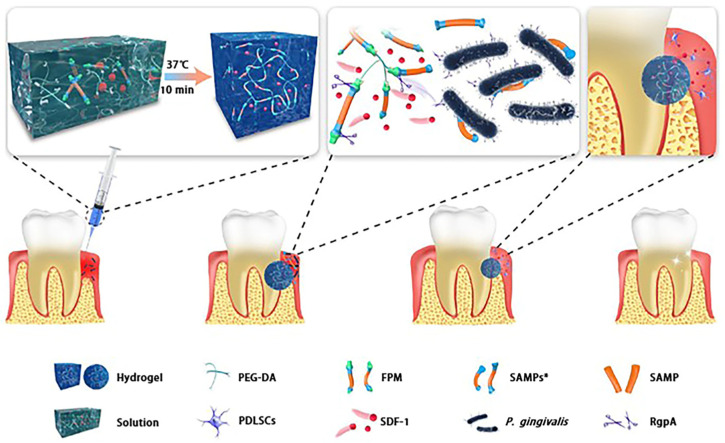
A schematic illustration depicting the process of preparing and utilizing multifunctional hydrogels. The thermosensitive hydrogel, which is responsive to gingivine, is crosslinked using PEG-DA and FPM, and subsequently loaded with SDF-1 for a duration of 10 min at a temperature of 37 °C. The FPM is composed of SAMP positioned in the center, flanked by two anchoring peptides on the periphery. Each anchoring peptide encompasses a splice site that is specific to RgpA. Upon contact with RgpA, released by *P. gingivalis* at the location of a periodontal defect, the hydrogel undergoes splicing at the designated site within the FPM, leading to the release of SAMPs*. Consequently, the growth of periodontal pathogens is inhibited. Reproduced from ref. [100]. Copyright 2021, with permission from American Chemical Society.

AMPs are naturally occurring short peptides predominantly possessing a cationic nature, which demonstrate a broad spectrum of antimicrobial activity [93]. The antimicrobial efficacy of AMPs stems mainly from the electrostatic interaction between the cationic peptide and the bacterial membrane. Binding of hydrophobic peptide components to the cell membrane leads to cell disruption, release of endogenous substances and rapid sterilization. As a result, the probability of bacterial resistance towards AMPs is considerably diminished [94,95,96,97]. AMPs, known for their limited resistance, also exhibit promising potential for utilization in the treatment of infected bones. Zhang et al. successfully applied a coating of GelMA hydrogels containing the antimicrobial peptide GL13K onto micro- and nanostructured titanium, resulting in biocompatible titanium-based materials. Furthermore, GL13K demonstrated controlled-release capabilities and displayed enhanced antimicrobial activity against both Gram-positive and Gram-negative bacteria [98]. Yang et al. employed the property of RADA16 self-assembling peptide to establish a stable structure, thereby facilitating the sustained release of AMPs for therapeutic purposes. Both in vitro and in vivo investigations substantiated that the RADA16-AMP self-assembling peptide not only exhibited superior inhibitory efficacy but also effectively promoted bone formation [99]. Conventional approaches for loading antimicrobial peptide hydrogels often lead to diffusive release of AMPs, which hampers the long-term precision of antimicrobial activity in the hydrogels. Liu et al. conducted a study in which they synthesized PEGPD@SDF-1 hydrogels using PEG diacrylate (PEG-DA) along with dithiothreitol (DTT) and functional peptide (FPM) through a Michael-type addition reaction. The FPM possesses a structure consisting of an anchor peptide, a short AMP (SAMP), and another anchor peptide. This structure allows for specific cleavage by gingival protease, resulting in the release of SAMP from the hydrogel in response to the antimicrobial effect of gingival protease. PEGPD@SDF-1 hydrogels demonstrated improved biocompatibility and promoted the proliferation, migration, and osteogenic differentiation of periodontal ligament stem cells (PDLSC) [100] (Figure 4). In summary, the combination of antimicrobial peptide and hydrogel is favorable for protecting the stability and delivery of AMPs, which makes it more effective in the treatment of infected bone.

### 3.2. Contact Sterilizing Hydrogels

Contact biocidal hydrogels offer a durable means of delivering biocidal substances by securely attaching them to the hydrogel, thereby avoiding any detrimental effects on the environment caused by the prolonged build-up of biocides. The biocidal properties of contact biocidal hydrogel surfaces are typically achieved through the incorporation of quaternary ammonium compounds, carbon nanotubes, or diverse cations.

#### 3.2.1. Natural Cationic Polymers

CS, a linear polysaccharide composed of d-glucosamine and n-acetyl-d-glucosamine and its derivatives, is the most abundant natural cationic polymer [101]. It has been extensively researched and utilized in the biomedical field for wound dressings, tissue engineering, and implantable coatings due to its notable antimicrobial activity and biocompatibility [102,103]. The antimicrobial mechanism of CS primarily relies on the protonation of its structural amino group in weakly acidic solutions, which disrupts bacterial cell membranes through electrostatic interactions, thereby inhibiting bacterial growth [104]. CS can be utilized in bone tissue engineering either in the form of a hydrogel or as a composite coating on implants to facilitate sterilization. Hydrogels, in particular, can establish a microenvironment that promotes bone repair, thereby expediting the healing process of infected bone defects [105,106].

**Figure 5:**
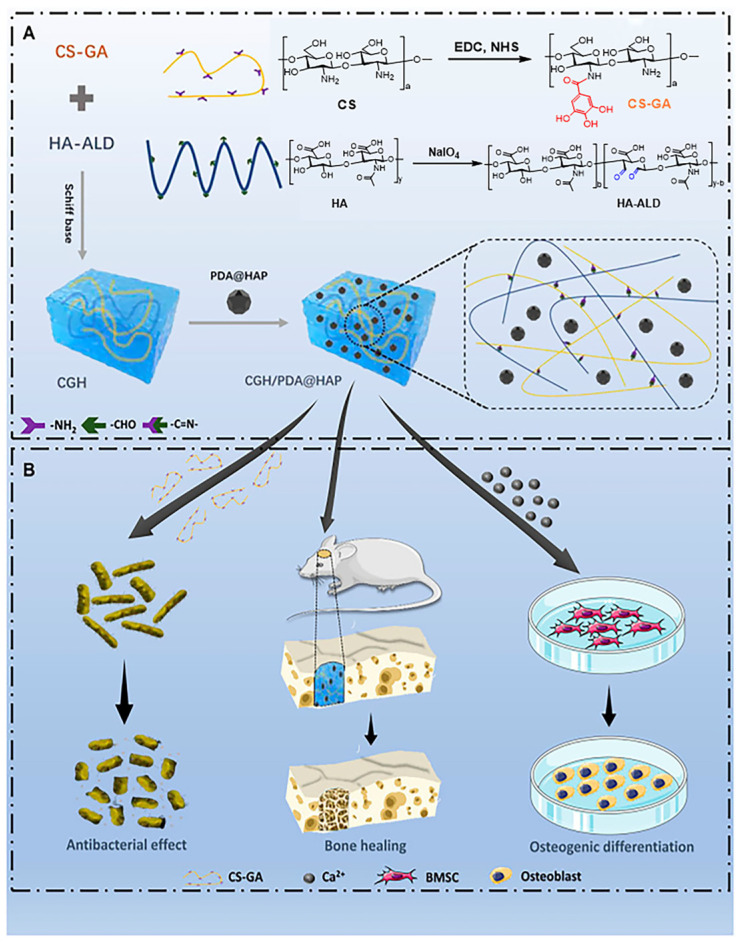
Preparation and biological effects of the CGH/PDA@HAP hydrogel. (**A**) Synthetic process. (**B**) Biological effects oxidized. (EDC: N-(3-Dimethylaminopropyl)-N′-ethylcarbodiimide hydrochloride, NHS: N-Hydroxysuccinimide, CS-GA: GA-modified CS, HA-ALD: oxidized HA). Reproduced from ref. [107]. under Creative Commons Attribution https://creativecommons.org/licenses/by/4.0/ (accessed on 13 December 2023). Copyright 2023. Pang et al.

In their study, Gilarska et al. developed a novel hydrogel composed of kynurenine cross-linked collagen, CS, and lysine-modified HA. This multifunctional hydrogel exhibited the ability to support osteoblast proliferation and adhesion, as well as promote ALP expression [61]. Furthermore, the researchers observed that the hydrogel with a higher CS content displayed significant antimicrobial activity. However, it was noted that the antimicrobial properties of CS are inherently limited but can be improved through modifications to enhance both its mechanical properties and antimicrobial effect. For example, Pang et al. conducted a study wherein they developed a composite hydrogel using gallic acid (GA), CS, and HA. By grafting GA onto CS, they successfully inhibited the growth of *Staphylococcus aureus* and *Escherichia coli*. Additionally, the hydrogels incorporating polydopamine (PDA)-modified HAP exhibited better potential in promoting the differentiation of BMSCs into osteoblasts, thereby displaying exceptional osteogenic activity [107] (Figure 5).

#### 3.2.2. Synthesis of Cationic Polymers

Cationic polymers, particularly those that are biodegradable, exhibit better antimicrobial efficacy and demonstrate a reduced propensity for bacterial resistance, rendering them highly promising antimicrobial agents in the treatment of infected bone [108]. The antimicrobial attributes of synthetic cationic polymers primarily rely on the presence of cationic and hydrophobic groups. Cationic groups have the potential to interact with negatively charged groups on the bacterial membrane, while hydrophobic groups can permeate the membrane and disrupt its integrity, ultimately leading to the demise of pathogenic bacteria [109,110]. The polymers exhibit a higher charge density compared to the monomers, thereby resulting in heightened antimicrobial activity. Additionally, incorporating longer alkyl chains or more hydrophobic aryl groups enhances the hydrophobic interactions with bacteria, consequently leading to increased antimicrobial activity [111,112]. Notably, cationic antimicrobial polymers, such as those containing quaternary ammonium groups, phosphorus groups, and biguanide groups, play a weighty role in synthetic antimicrobial agents.

**Figure 6:**
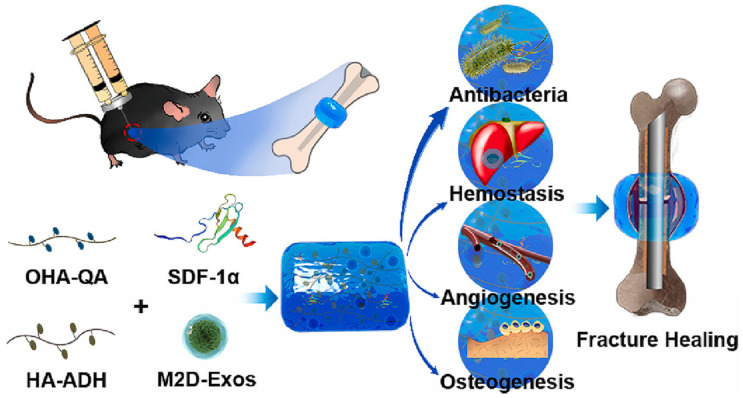
The mechanisms underlying HA@SDF-1α/M2D-Exos hydrogel’s ability to accelerate fracture healing. The HA@SDF-1α/M2D-Exos hydrogel can be constructed in situ through a mixed injection process. The hydrogel forms rapidly due to the hydrazone bond formation between the HA-ADH and the OHAQA crosslinking, while the positively charged quaternary ammonium groups of the hydrogel provide a long-term antibacterial and hemostasis environment. Synchronously and sustainably released SDF-1α and M2D-Exos from the HA@SDF-1α/M2D-Exos hydrogel enhance osteogenesis and angiogenesis both in vivo and in vitro. Reproduced from ref. [113]. Copyright 2023, with permission from Elsevier.

Quaternary ammonium salts possess broad spectral antimicrobial properties and can disrupt microbial biofilms through hydrophobic and ionic interactions. Chen et al. employed oxidized HA-quaternary ammonium salt (OHA-QA) and adipic dihydrazide-modified HA (HA-ADH) in the synthesis of injectable, self-repairing, and mucoadhesive HA@SDF-1α/M2D-Exos hydrogels. The positively charged quaternary ammonium groups of the hydrogels created a sustained antibacterial and hemostatic environment. Additionally, the controlled and continuous release of SDF-1α and M2D-Exos from the HA@SDF-1α/M2D-Exos hydrogels promoted osteogenesis and angiogenesis both in vivo and in vitro [113] (Figure 6).

## 4. Bone Repair Hydrogels

The improvement of infection control efficacy and bone defect repair functionality is imperative for the development of infected bone therapy hydrogels. Bone regeneration is facilitated by the interplay of cytokines and bioactive substances, which regulate cellular interactions within their surrounding environment [114,115]. Effective delivery of bioactive substances to bone defects can facilitate the repair process by transporting drugs, cells, and growth factors. Consequently, the design of a drug delivery system enables localized delivery, minimizing adverse effects on adjacent tissues and organs [116,117,118].

### 4.1. Cells

The repair process of bone defects consists of fundamental multicellular units, namely osteoblasts, and osteoclasts [119,120]. In the clinical management of bone defects, cell-based therapy represents one of the more efficacious approaches for bone repair [121]. Stem cell therapy primarily entails the injection of stem cell suspensions into bone defects using syringes; nevertheless, the microenvironment within the bone defect hinders the sustenance of sufficient quantities and viability of stem cells. Implanting a hydrogel as a carrier for cells into bone defects is a recommended strategy to create an optimal cellular microenvironment that facilitates cell adhesion, value addition, and differentiation. In the field of bone repair studies, the use of BMSCs is prevalent [122,123,124]. BMSCs possess the ability to self-renew and differentiate, along with the secretion of paracrine mediators and trophic factors, immunomodulation, angiogenesis, and better osteogenic potential [125,126].

**Figure 7:**
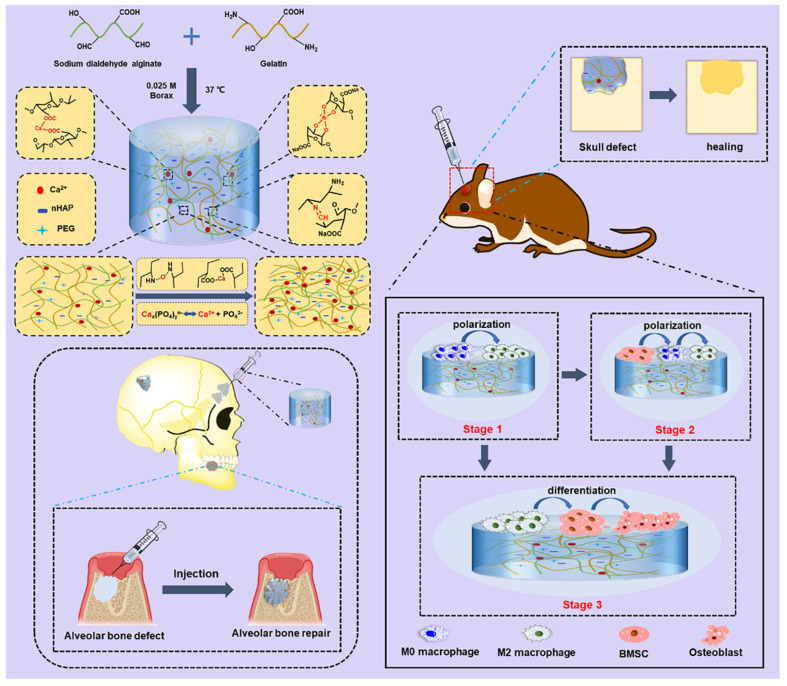
Schematic diagram of a composite ADA−Gel hydrogel double cross-linked by Ca^2+^ and chemically bonded and decorated with nHAP. Dynamic dissociated PO_4_^3−^ and Ca^2+^ from nHAP interact with the biopolymer to form a tight and compact structure. Improved biomedical and mechanical properties were achieved for its application in maxillofacial bone defects. Through interactive modulation between macrophages and BMSCs, the composite hydrogel significantly accelerated the bone repair process. Reproduced from ref. [129]. Copyright 2022, with permission from Elsevier.

Li et al. prepared hydrogel scaffolds using bionic methacrylated gelatin (Bio-GelMA) and encapsulated BMSCs. These Bio-GelMA scaffolds exhibited biocompatibility and demonstrated chemical and physical similarities to the natural bone extracellular matrix. In comparison to hydrogel and stem cell scaffolds, the Bio-GelMA hydrogel scaffolds loaded with BMSCs yielded the most favorable outcomes in terms of new bone formation in a rat segmental bone defect model [127]. On the other hand, Liu et al. demonstrated that the incorporation of nano-attrapulite (nano-ATP) into GelMA hydrogel resulted in enhanced cell-material interaction and improved mechanical strength, thereby effectively promoting bone regeneration [128]. Additionally, the structural characteristics of hydrogels are crucial in the context of bone repair. Zhou et al. successfully developed an injectable double-crosslinked hydrogel based on gelatin, employing covalent crosslinking of alginate dialdehyde and ionic crosslinking of Ca^2+^. The composite hydrogels exhibited improved compressive tensile strength and shape adaptability, making them suitable for effectively covering irregular bone defects. The composite hydrogels were fabricated by incorporating nHAP into highly ordered, uniform, and size-restricted bioactive porous structures. This integration enabled effective communication and interaction between macrophages and BMSCs, creating an interactive osteogenic platform [129] (Figure 7). Furthermore, other cell-based bone tissue engineering approaches have also demonstrated comparable osteogenic capabilities. For example, Wang et al. conducted a study wherein they created an innovative injectable calcium phosphate bone cement scaffold. This scaffold incorporated hydrogel fibers that encapsulated various types of stem cells, including human induced pluripotent stem cell-derived mesenchymal stem cells (hiPSC-MSCs), human BMSCs, and human premolar dental pulp stem cells (hDPSCs). The researchers then proceeded to compare the viability, proliferation, and osteogenic differentiation of these cells [130]. The findings indicated that the hydrogel fibers within the calcium phosphate bone cement scaffold facilitated the proliferation and differentiation of multiple cell types into osteogenic lineages. These results are highly promising and hold great potential for the field of bone tissue engineering.

### 4.2. Growth Factors

Growth factors, which are active proteins or peptide substances, play a crucial role in regulating the growth and development of organisms [131]. The process of bone defect healing involves a signaling cascade, wherein cells at the defect site secrete growth factors, cytokines and other substances, leading to the recruitment of surrounding osteoblasts for migration, proliferation, and differentiation [132]. Given the weighty reliance of the bone healing mechanism on bioactive factors, the introduction of exogenous bioactive factors through injection can expedite the process of bone repair [133]. Nevertheless, the majority of bioactive factors are proteins, and their direct delivery is prone to degradation by enzymes [134]. Hence, appropriate carriers must be used to ensure safe delivery of the active factors and to prevent their inactivation. It is also important to facilitate precise delivery of growth factors and reduce rapid diffusion. Failure to do so may lead to inflammatory reactions and other adverse effects [135]. Hydrogels possess a distinctive architecture that enables the controlled release of bioactive factors in both spatial and temporal dimensions, thereby facilitating their sustained release over an extended duration. Consequently, this sustained release mechanism facilitates the continuous formation of bone matrix and blood vessel networks [136,137]. Hydrogels also possess the characteristics of water absorption and swelling, thereby creating a hydrophilic environment that effectively prevents factor inactivation. Additionally, hydrogels can serve as a biomimetic extracellular matrix (ECM), making them exceptional carriers for maintaining factor activity [138]. Among the extensively studied growth factors in the realm of bone regeneration, bone morphogenetic protein (BMP) [139], vascular endothelial growth factor (VEGF) [140], insulin-like growth factor (IGF) [141] and fibroblast growth factor (FGF) [142] have all demonstrated weighty potential in enhancing bone regeneration.

**Figure 8:**
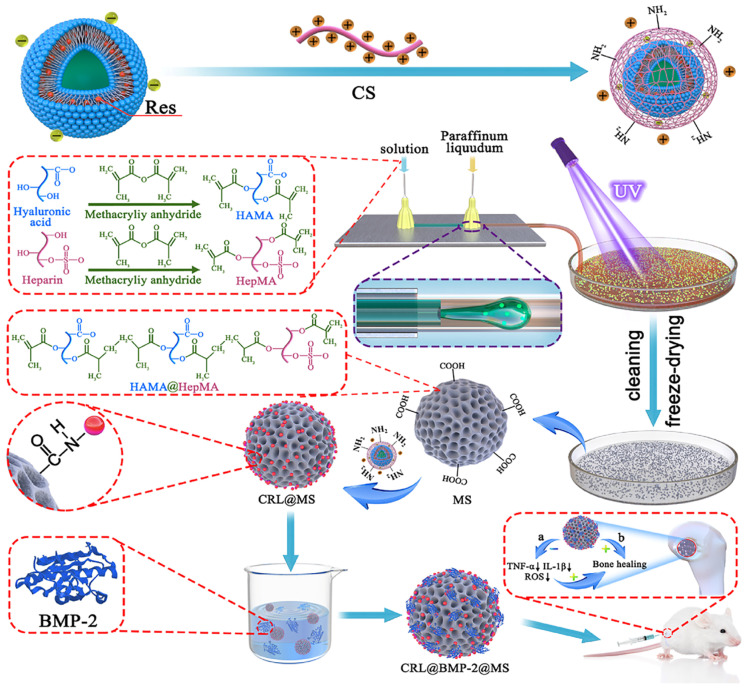
Composite schematic diagram of CRL@ BMP-2@MS. Reproduced from ref. [145]. under Creative Commons Attribution https://creativecommons.org/licenses/by/4.0/ (accessed on 13 December 2023). Copyright 2023. Cai et al.

BMP, a member of the superfamily of transforming growth factors beta, serves as a signaling molecule for the process of mineral deposition and the differentiation of pluripotent cells into osteoblasts [143]. Notably, BMP-2 exhibits notable osteoinductive properties and plays a vital role in regulating bone morphogenesis. Despite the promising bone repair effects demonstrated by therapeutic agents like BMP-2 in diverse clinical scenarios, it is important to consider the issue of uncontrolled release kinetics. Xu et al. employed electrostatic interaction to immobilize the BMP-2 side chain with mineral-coated micromeres (MCMs), which were subsequently anchored into hydrogels through cross-linking of CS and PEG. The resulting particulate hydrogel system exhibited sustained release of BMP-2, demonstrated good biocompatibility, and facilitated excellent vascularization in bone defects [144]. Moreover, Cai utilized CS liposomes CS-Res@Lipo (CRL), which were prepared using the film dispersion method and static loading technique, along with HAMA@HepMA hydrogel microspheres (MS), which were prepared using microfluidics, for a condensation reaction chemical grafting process. BMP-2 was bound to the non-covalent binding sites of the MS microspheres, resulting in the construction of programmed-release hydrogel microspheres (CRL@BMP-2@MS) (Figure 8). These CRL@BMP-2@MS microspheres were found to regulate acute bone immune myopathies through the early release of Res, while the sustained release of BMP-2 facilitated the programmed synergistic linkage between immunity and osteogenesis, thereby dynamically regulating bone repair. This novel approach offers a promising avenue for further exploration in the repair of large bone defects [145]. In contrast, Mao et al. conducted a study in which they covalently integrated BMP-2 with silver core-embedded mesoporous silica nanoparticles (Ag@MSN) and subsequently encapsulated them within Filipino methacryloyl hydrogel. This approach effectively preserved the bioactivity of BMP-2 while also prolonging its release. Moreover, the inclusion of Ag in the hydrogel induced antimicrobial properties, leading to a synergistic effect that promoted osteoblastogenesis and exhibited antimicrobial activity [146].

VEGF, a crucial molecule governing the growth of bone, cartilage, vascular tissues, and angiogenesis, was employed by Elango to fabricate collagen-CS hydrogels (HGs) with varying concentrations. The objective of the study was to investigate the impact of these HGs on the proliferation, structure, and osteogenic potential of human bone marrow-derived mesenchymal stem cells (hBMMSCs). The findings revealed that diverse concentrations of VEGF expedited MSC proliferation and augmented osteogenic stimulation when combined with osteogenic supplements. HG loaded with VEGF accelerated the deposition of mineral and ALP levels in osteoblasts and improved the osteogenesis of MSCs when osteogenic inducers were present [147].

**Figure 9:**
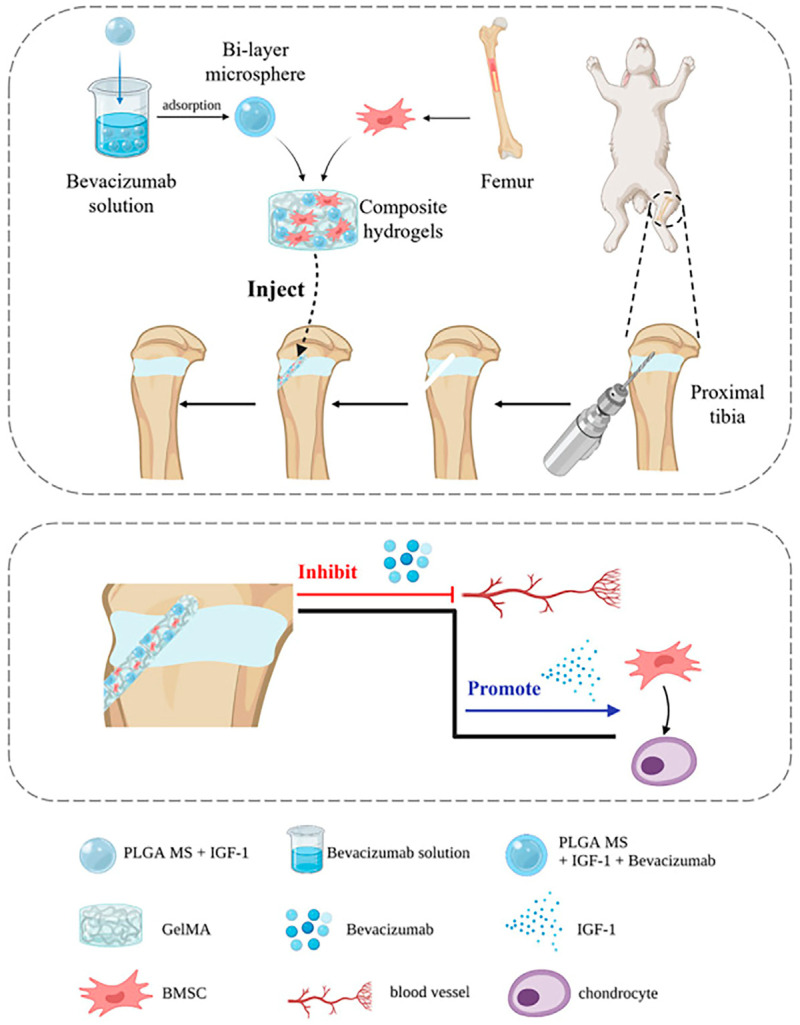
Schematic illustration of the study design of a composite hydrogel consisting of GelMA and BMSCs and wrapped with a two-layer drug-loaded poly (lactic-co-glycolic acid) (PLGA) microsphere system consisting of bevacizumab and IGF-1 to achieve sustained drug delivery to the injury site. Reproduced from ref. [148] under Creative Commons Attribution https://creativecommons.org/licenses/by/4.0/ (accessed on 13 December 2023). Copyright 2023. Qiang et al.

Qiang et al. successfully synthesized a composite hydrogel that exhibited favorable injectability and biocompatibility through the integration of a bilayer drug-carrying microsphere. The external layer of the microsphere was loaded with bevacizumab, a potent inhibitor of vascular endothelial growth factor, while the internal layer contained IGF-1. This microsphere was then incorporated into a gelatinized methacrylamide hydrogel alongside BMSCs. The bilayer microspheres functioned as an efficient drug delivery system, releasing bevacizumab first, followed by IGF-1 in a sequential manner. This hydrogel exhibited simultaneous inhibition of angiogenesis and promotion of cartilage regeneration [148] (Figure 9).

FGF is a growth factor that exhibits the ability to be readily incorporated into hydrogels. For instance, Teng et al. successfully incorporated KGN and FGF-2 into a mercapturized CS-based hydrogel which demonstrated efficacy in the controlled release and targeted delivery of both factors to the site of injury, thereby facilitating prompt healing at the interface between the tendon and bone [149].

## 5. Conclusions

The treatment of infected bone defects remains a formidable challenge in orthopedics, oral implantology, and maxillofacial surgery. Given the limited effectiveness of current clinical interventions, there is a growing interest in the development of novel hydrogels that possess both antimicrobial and osteoinductive properties. These innovative hydrogels exhibit enhanced biocompatibility, diminished resistance, and a wider range of antimicrobial activity compared to the conventional use of antibiotics in clinical practice. Furthermore, the widespread use of bone repair drugs provides a viable treatment for infected bone repair. Among them, BMP, especially BMP-2, is currently recognized as the most effective osteoinductive drug, stimulating osteogenesis in vitro and in vivo. Treatment of infected bone usually requires the integration of antimicrobials and osteoinductive drugs into a co-delivery system. This system facilitates the release of antimicrobial and osteoinductive drugs in different patterns and exhibits different kinetic and temporal characteristics. This review can inform future research on hydrogel drug delivery systems. This includes refining the stimulus responsiveness of hydrogels and improving targeted drug delivery mechanisms. Thus, allowing the drug to be delivered to a specified location under controlled conditions. Also, improving the controlled release of hydrogel drugs is improved, using the correct and precise kinetics to release them. This enables the clinical application of innovative hydrogels with antimicrobial and bone repair-promoting properties to have a broader outlook.

At present, the clinical results of hydrogel-based infected bone repair are still not up to satisfactory standards and the treatment of large bone defects remains a major challenge. Natural biodegradable materials such as CS, HA and SA have good biocompatibility and tissue repair properties. However, they suffer from low mechanical strength and uncontrolled degradation, whereas hydrogels more adapted to bone repair can be formed by compounding with other materials. Synthetic hydrogel polymers have some disadvantages although they have the advantages of slow degradation, high immunogenicity and limited biocompatibility. Therefore, future hydrogel research should focus more on clinical applications. This includes the development and application of novel antimicrobial hydrogels to avoid the increase of multidrug-resistant bacteria and underdosing. Although progress has been made in the osteogenic properties of natural and synthetic hydrogels, their angiogenic capacity as well as their practical applicability should not be overlooked. There is a need for more comprehensive studies to optimize the porosity, strength and drug-carrying release of hydrogels. This leads to the formation of engineered bones adapted to in vivo conditions from implantation to bone reconstruction.

## Figures and Tables

**Table 1:** Advantages and limitations of hydrogel materials.

Hydrogel Types	Hydrogel Materials	Advantages	Limitations	References
Natural Hydrogels	Chondroitin sulfate	Modulation of bone remodeling	High solubility under physiological conditions	[46,47,48]
	Hyaluronic acid	Tissue healing and angiogenesis	Requires modification to form stable hydrogels	[49,50]
	Chitosan	Antimicrobial properties, controlled degradability, biocompatibility	Poor mechanical properties	[51,61]
	Sodium alginate	Biocompatible	Poor cell adhesion and poor mechanical properties	[52]
Synthetic Hydrogels	Polyvinyl alcohol	Water soluble, biocompatible	Poor mechanical properties	[56]
	Polycaprolactone	Elastic, thermally stable, flexible, and biocompatible	Slow degradation speed	[57]
	Polyethylene glycol	Biocompatible, low toxicity, anti-cell protein adhesion	Moisture sensitivity	[59,60]

## Data Availability

Not applicable.
